# Clinical Outcomes of RTOG 9310 Protocol for Primary Central Nervous System Lymphoma: Single-Center Experience with 87 Patients

**DOI:** 10.3390/curroncol28060393

**Published:** 2021-11-12

**Authors:** Jinuk Kim, Tae Gyu Kim, Hyoun Wook Lee, Seok Hyun Kim, Ji Eun Park, Moonok Lee, Young Zoon Kim

**Affiliations:** 1 Division of Neuro Oncology, Department of Neurosurgery, Samsung Changwon Hospital, Sungkyunkwan University School of Medicine, Changwon 51353, Korea; jinukstar@gmail.com; 2 Department of Radiation Oncology, Samsung Changwon Hospital, Sungkyunkwan University School of Medicine, Changwon 51353, Korea; ktg7757@skku.edu; 3 Department of Pathology, Samsung Changwon Hospital, Sungkyunkwan University School of Medicine, Changwon 51353, Korea; sudowo@skku.edu; 4 Division of Hematology and Medical Oncology, Department of Internal Medicine, Samsung Changwon Hospital, Sungkyunkwan University School of Medicine, Changwon 51353, Korea; tjrgus1@hanmail.net; 5 Department of Anesthesiology and Pain Medicine, Samsung Changwon Hospital, Sungkyunkwan University School of Medicine, Changwon 51353, Korea; jnpark369@gmail.com (J.E.P.); lmo602@hanmail.net (M.L.)

**Keywords:** adverse effect, chemotherapy, methotrexate, primary CNS lymphoma, prognosis

## Abstract

The Radiation Therapy Oncology Group (RTOG) 9310 protocol clinical trial established high-dose methotrexate (HDMTX) as the standard for primary central nervous system lymphoma (PCNSL). We aimed to investigate the RTOG 9310 protocol’s PCNSL outcomes by examining progression-free survival (PFS) and overall survival (OS) rates and determining the influential factors. Between 2007 and 2020, 87 patients were histopathologically diagnosed with PCNSL and treated with the RTOG 9310 protocol. All received HDMTX 2.5 g/m^2^ and vincristine 1.4 mg/m^2^/day for 1 day during weeks 1, 3, 5, 7, and 9, and procarbazine 100 mg/m^2^/day for 1 day during weeks 1, 5, and 9. Dexamethasone was administered on a standard tapering schedule from the first week to the sixth week. Whole brain radiotherapy (WBRT), consisting of 45 Gy for patients with less than a complete response to the chemotherapy or 36 Gy for complete responders, was started 1 week after the last dose of chemotherapy was administered. Within three weeks of the completion of WBRT, patients received two courses of cytarabine, which were separated by 3–4 weeks. Clinical, radiological, and histopathological characteristics were retrospectively reviewed. All patients completed five HDMTX cycles and a mean follow-up of 60.2 (range, 6–150) months. Twenty-eight (32.2%) patients experienced recurrence during follow-up. The mean time to recurrence was 21.8 months, while the mean PFS was 104.3 (95% confidence interval (CI), 90.6–118.0) months. Eleven (12.6%) patients died; the mean OS was 132.1 (95% CI, 122.2–141.9) months. The 3- and 5-year survival rates were 92.0% and 87.4%, respectively. One patient experienced acute renal failure, while the remainder tolerated any cytotoxic side effects. On multivariate analysis, the Eastern Cooperative Oncology Group performance score ≤ 2; the International Extranodal Lymphoma Study Group low-risk status; XBP-1, p53, and c-Myc negativity; homogenous enhancement; gross total resection, independently correlated with long PFS and OS. The RTOG 9310 protocol is effective for PCNSL and features good outcomes.

## 1. Introduction

Primary central nervous system lymphoma (PCNSL) is a highly aggressive extranodal subtype of non-Hodgkin lymphoma that is usually confined to the brain, eyes, leptomeninges, or spinal cord in the absence of systemic lymphoma. More than 95% of PCNSL cases have a histology comparable to that of diffuse large B-cell lymphoma (DLBCL) [1]. PCNSL are rare tumors that account for up to 1% of all lymphomas, 4–6% of all extranodal lymphomas, and approximately 3% of all primary brain tumors worldwide, including Korea [2]. Immunocompromised individuals such as those with human immunodeficiency virus (HIV) and Epstein–Barr virus (EBV) infections are considered most at risk of developing PCNSL; however, its incidence is increasing in immunocompetent populations and now represents the vast majority of patients [3,4,5]. PCNSL typically follows an aggressive course and, despite treatment advances, remains associated with very high mortality rates [1].

Although PCNSL is a highly malignant tumor with a poor prognosis in untreated patients in contrast to most malignant brain tumors, it is sensitive to corticosteroids, chemotherapy, and radiotherapy, which can achieve complete remission (CR) and extended long-term survival. For systemic non-Hodgkin lymphoma, the cyclophosphamide, doxorubicin, vincristine, and prednisolone regimen is routinely used and induces responses with relatively good outcomes. However, this regimen is not recommended for PCNSL because it offers no survival advantage over radiotherapy alone [6,7,8]. The apparent inability of these chemotherapeutic regimens to cross the brain–blood barrier and eradicate microscopic disease is a major challenge. To date, intravenous high-dose methotrexate (HDMTX) is considered the most important and beneficial single drug for PCNSL based on convergent results from many prospective and retrospective studies [1,9,10]. Although HDMTX is the backbone for the treatment of newly diagnosed PCNSL, neither a concrete combination regimen nor an effective dose has been conclusively established. Controversy persists about which type of consolidation regimen may be most beneficial after HDMTX-based induction therapy.

Whole brain radiotherapy (WBRT) was used to treat newly diagnosed PCNSL until the early 1980s, and the introduction of HDMTX plus WBRT improved clinical outcomes with a median overall survival (OS) of 30–60 months and 5-year survival rates of 30–50% [11]. Rituximab, a monoclonal antibody directed against the B-cell surface antigen CD20, recently, dramatically improved the response and clinical outcomes of DLBCL and was incorporated into first-line PCNSL treatment regimens. The therapeutic outcome has improved substantially in the past two decades as a result of better curative treatment strategies. However, the treatment of this disease remains challenging because remission is frequently of short duration and recurrence is not prevented.

The Radiation Therapy Oncology Group (RTOG) 9310 protocol of combination chemotherapy composed of HDMTX, procarbazine, and vincristine followed by radiotherapy for PCNSL is a traditional regimen with good therapeutic outcomes [12,13]. Their report of the first multicenter trial demonstrated improved survival with the combination of chemotherapy and radiotherapy compared with previous reports of radiotherapy alone. They showed that an HDMTX-based regimen produced a high response rate before radiotherapy was administered and HDMTX plus cranial irradiation effectively treated PCNSL. As neurotoxicity was considered a delayed risk of the RTOG 9310 protocol, the dose of radiotherapy was reduced from 45 Gy/25 fractionation to 36 Gy/30 fractionation in a secondary analysis [13]. This analysis showed that progression-free survival (PFS) and OS rates were not significantly affected despite the hyperfractionated radiotherapy schedule representing a 25% reduction in biologically effective tumor dose [13].

After the report of improved clinical results from the RTOG 9310 protocol for PCNSL patients was published in 2005, we started to treat newly diagnosed PCNSL patients using this protocol at our institute. In the present study, we primarily investigated the PFS and OS of PCNSL patients treated with the RTOG 9310 protocol. We also examined the predictive factors associated with PFS and OS to be validated with previously known prognostic factors in the literature. This analysis included pathological markers that were not addressed in the previous RTOG study for predicting clinical outcomes. Several tips for the application of the RTOG 9310 protocol from our experience are discussed to reduce the adverse effects and improve the treatment success.

## 2. Materials and Methods

### 2.1. Patient Enrolment

Newly diagnosed immunocompetent patients with PCNSL who were treated at our institute between March 2007 and August 2020 were evaluated for study participation. Inclusion and exclusion criteria were applied as in the RTOG 9310 protocol [12]. The inclusion criteria were as follows: histologic proof of non-Hodgkin lymphoma by brain biopsy; histological diagnosis received within the previous 4 weeks; absolute neutrophil count (ANC) ≥ 2000 cells/mm^3^; platelets ≥ 100,000 cells/mm^3^; total bilirubin ≤ 2.0 mg; serum glutamic oxaloacetic transaminase (SGOT) ≤ 2 times the upper limit of normal; creatinine clearance ≥ 50 cc/min/1.73 m^2^; normal serum electrolytes; a life expectancy of at least 8 weeks; Eastern Cooperative Oncology Group (ECOG) performance score ≤ 3; HIV-1 negative serology. The exclusion criteria were as follows: prior cranial irradiation; concurrent malignancies; pre-existing immunodeficiency such as renal transplantation; prior treatment with chemotherapy; ECOG performance score ≥ 4; ANC < 2000 cells/mm^3^; platelets < 100,000 cells/mm^3^; total bilirubin > 2.0 mg; SGOT > 2 times the upper limit of normal; creatinine clearance < 50 cc/min/1.73 m^2^; abnormal serum electrolytes; currently pregnant or nursing. Patients who were excluded from this protocol were scheduled to undergo radiotherapy alone or supportive care.

To exclude evidence of systemic lymphoma, a negative staging evaluation using chest, abdominal, and pelvic computed tomography and bone marrow biopsy was required. All patients underwent cranial neuroimaging at the time of diagnosis, preferably using magnetic resonance image (MRI). All patients underwent lumbar puncture and complete ophthalmologic evaluation, including a slit-lamp examination. The ECOG performance status definition was used [14]. Before chemotherapy, a laboratory diagnosis of EBV infection was made on a single serum sample using a standard immunofluorescence test for antibodies to EBV-associated antigens, simultaneously; immunoglobulin M and G to the viral capsid antigen, the early antigen, and the EBV nuclear antigen.

### 2.2. Treatment Protocol

The protocol was performed as previously reported by the RTOG [12]. Chemotherapy was administered for five cycles over a 10-week period (Supplementary Table S1). Each cycle consisted of methotrexate 2.5 g/m^2^ infused over 2–3 h and vincristine 1.4 mg/m^2^ with a cap of 2.8 mg. Methotrexate was followed by vigorous hydration at a rate of 1500–1800 mL/m^2^ for the first 24 h, followed by a rate of 2000 mL/m^2^ for the subsequent 48 h. Urine alkalinization was accomplished with intravenous bicarbonate, and rescue leucovorin 20 mg orally every 6 h for 12 doses was initiated 24 h after methotrexate administration. In addition to methotrexate and vincristine, procarbazine 100 mg/m^2^/day for 7 days was administered during cycles 1, 3, and 5. All patients with positive CSF findings underwent Ommaya reservoir placement and the administration of methotrexate 12 mg for five cycles the week after each dose of intravenous methotrexate. Intra-Ommaya methotrexate was followed by oral leucovorin 10 mg every 6 h for eight doses immediately after each dose of intrathecal MTX administration. Dexamethasone was administered on a standard tapering schedule of 16 mg/day for the first week, 12 mg/day for week 2, 8 mg/day for week 3, 6 mg/day for week 4, 4 mg/day for week 5, and 2 mg/day for week 6; however, the dose could be adjusted according to the patient’s neurological condition. All patients received trimethoprim/sulfamethoxazole prophylaxis for *Pneumocystis carinii* pneumonia.

WBRT was planned for a total dose of 45 Gy in 1.80-Gy fractions. If ocular lymphoma was present, both eyes were included in the radiotherapy field to a total dose of 36 Gy in 20 fractions. Approximately halfway through the study, there was growing evidence from the RTOG report that long-term survivors of combined methotrexate-based chemotherapy and cranial irradiation developed permanent severe neurotoxicity [12]. This is a particularly significant issue for older patients. Our institute’s protocol was then modified so that those patients who achieved a CR at the end of 10 weeks of chemotherapy would receive a reduced dose of WBRT course for a total dose of 36 Gy given in 1.8-Gy fractions for 20 days, which was started 1 week after the last dose of chemotherapy was administered [12].

At the completion of cranial irradiation, all patients were scheduled to receive two courses of high-dose cytarabine within three weeks of the completion of WBRT according to the original RTOG protocol. Each course consisted of two doses separated by 24 h of cytarabine 3 g/m^2^/day infused over 3 h (Supplementary Table S1).

When the disease recurred or progressed, repeated HDMTX-based combined chemotherapy regimen was used based on the initial RTOG 9310 protocol, and was considered primarily without brain irradiation.

### 2.3. Clinical Data Collection

Epidemiological characteristics (including sex, age at the time of the PCNSL diagnosis, and performance status), type of primary treatment for PCNSL, type of salvage treatment for recurrent and progressive disease (PD), duration of follow-up, and time of death were retrospectively reviewed from the medical records of each patient. For prognostic assessment, additional factors, such as the serum lactate dehydrogenase (LDH) level, protein concentration in the cerebrospinal fluid (CSF), and deep brain involvement, were evaluated using the literature criteria provided by International Extranodal Lymphoma Study Group (IELSG) [15]. All patients were treated with the same protocol of high-dose methotrexate-based combination chemotherapy followed by adjuvant WBRT.

The radiological characteristics of the brain lesions were evaluated using MRI at the time of the initial diagnosis of PCNSL. The number was classified as unifocal or multifocal based on whether the mass was enhanced with gadolinium on T1-weighted MRI. The basal ganglia, corpus callosum, brain stem, and cerebellum were defined as deep brain structures. Peritumoral edema was categorized as < or ≥2 cm from the brain tumor as assessed by T2-weighted MRI. In cases of multiple brain lesions, regardless of their number, different patterns of enhancement on MRI were considered heterogeneous enhancement. The radiological evaluation was performed by two different neuroradiologists (Y.M. Kim, Samsung Changwon Hospital, Changwon, Korea and M.O. Sunwoo, Samsung Changwon Hospital, Changwon, Korea) who were blinded to the clinical and pathological parameters.

In terms of histopathological characteristics, a routine analysis of the diagnostic markers was performed at the time of the initial diagnosis, such as pathological diagnosis according to the 2016 World Health Organization classification [16]; cell type; EBV in situ hybridization; Ki67 index; BCL2 or BCL6, p53, MUM1, and c-Myc immunoreactivity. According to the literature, the cutoff value for meaningful immunoreactivity was defined as “positive” immunohistochemical staining of at least 40% of the tumor cells for c-Myc and 30% for BCL2 or BCL6 [17]. These features were obtained from pathological reports without additional immunohistochemical staining.

### 2.4. Follow-Up

Treatment response was assessed by contrast-enhanced brain MRI performed no more than 7 days before the commencement of chemotherapy, after the second and fifth HDMTX chemotherapy cycles, and 4 weeks after the termination of chemotherapy. The National Cancer Institute standardized response criteria regarding changes in enhanced lesion size on T1-weighted MR images were used to define treatment response [18]. In brief, a CR was defined as the complete disappearance of all evidence of lymphoma, partial response (PR) as a ≥50% decrease in tumor size, PD as a ≥25% increase in tumor size or the appearance of any new lesion, and stable disease (SD) as none of the above. Progression was defined as a new lesion with enhancement on MRI or an increase in tumor size by 25% or more. The time to progression and PFS were calculated from the diagnosis of PCNSL.

Repeat neuroimaging was required after the completion of neoadjuvant chemotherapy and radiotherapy, every 2 months for 1 year, every 4 months for the second year, and every 6 months thereafter. A comprehensive review of the chemotherapy records was performed to assess protocol adherence, and a radiologic review was performed by two neuroradiologists to assess the response to neoadjuvant chemotherapy as mentioned above. All adverse effects were recorded during the follow-up period according to the Common Terminology Criteria for Adverse Events provided by the National Cancer Institute [19].

### 2.5. Survival and Statistical Analyses

The medical records of the clinical history and radiographic reports of all study subjects were analyzed. The date of death was confirmed and recorded. OS was defined as the time from the date of the histological diagnosis of PCNSL until death. The date of biopsy or surgical resection of the brain lesion was recorded as the date of diagnosis.

The statistical analyses were performed using SPSS ver. 20.0 (IBM Corp., Armonk, NY, USA). Differences between subgroups were analyzed with Student’s t-test for normally distributed continuous values, the Mann–Whitney U test for non-normally distributed continuous values, and chi-squared tests for categorical variables. OS and PFS were calculated using the Kaplan–Meier method. Comparisons among groups were performed using the log-rank test. Variables that were significantly associated with a longer OS and PFS on the univariate analyses were examined using multivariate analysis. Several additional variables associated with OS and PFS in the literature and of interest to the investigators were also included in the multivariate analysis. In this analysis, the Cox proportional hazards regression model was used to assess the independent effects of specific factors on OS and PFS and define the hazard ratios of the significant covariates. Differences were considered statistically significant at two-sided values of *p* < 0.05.

As there is no universal cutoff value for the several clinical factors that predict OS of PCNSL, receiver operating characteristic curve analysis and a sensitivity–specificity analysis were used to define the cutoff value for the pathological biomarkers BCL2, BCL6, p53, MUM1, and c-Myc as predictive factors for OS of PCNSL. Through the sensitivity–specificity analysis, the cutoff value (the point at which sensitivity and specificity intersect) was determined for each value as correlated with survival [20].

## 3. Results

### 3.1. Patient and Tumor Characteristics

Among 100 patients who were newly diagnosed with PCNSL through brain biopsy or resection between March 2007 and August 2020, eight patients refused chemotherapy and underwent radiotherapy alone. Another five patients underwent supportive care alone instead of the HDMTX-based combination chemotherapy because they did not meet the eligibility criteria, for instance, three had an ECOG performance score of 4 and two had creatinine clearance <50 cc/min/1.73 m^2^. Ultimately, 87 patients were enrolled in the study after 13 patients were excluded. The study population included 45 men (51.7%) and 42 women (48.3%). The mean age of these patients at the time of the PCNSL diagnosis was 57.6 years (range, 32.4–81.2 years). Six patients (6.9%) had ocular tumor involvement. Serum lactate dehydrogenase (LDH) levels were elevated in 32 patients (36.8%), while cerebrospinal fluid (CSF) protein concentrations were elevated in 54 patients (62.1%). Sixty-two patients (71.3%) exhibited an independent performance status in daily activity with an Eastern Cooperative Oncology Group (ECOG) performance score of 0–1, while 25 patients (28.7%) exhibited a score of 2–3. In terms of risk evaluation for prognosis, 28 patients (32.2%) were categorized as low risk (International Extranodal Lymphoma Study Group (IELSG) score of 0–1), 24 (39.1%) as intermediate risk (IELSG score of 2–3), and 25 (28.7%) as high risk (IELSG score of 4–5) (Table 1). There was a significant difference in recurrence rate according to the ECOG performance score (0–1 versus 2–3; *p* = 0.037) and the risk group (*p* = 0.048) (Table 1).

A radiological analysis showed 57 (65.5%) unifocal lesions, 38 (43.7%) cases of deep brain involvement, and five (5.7) cases of CSF seeding. The maximal tumor size was ≥3 cm in 40 (46.0%) patients and peritumoral edema was ≥2 cm in 33 (37.9%) patients. Homogeneous enhancement was observed in 19 (21.8%) patients versus heterogeneous enhancement in 68 (78.2%) patients. Thirty-one (35.6%) patients underwent gross total resection, while the other 56 (64.4%) underwent partial resection or brain biopsy (Table 2). There was a significant difference in the recurrence rate according to the enhancement pattern (homogenous versus heterogeneous; *p* = 0.042) and surgical extent (gross total versus partial resection/biopsy; *p* = 0.036) (Table 2).

In terms of histopathological features, there were 74 (85.1%) cases of DLBCL and 32 (36.8%) cases of germinal center B-cell-like lymphoma. The cutoff values of each pathological biomarker for determining immunohistochemical positivity and negativity were as follows: 30% for BCL2 (area under the curve (AUC), 0.688; sensitivity, 72.3%; specificity, 65.4%), 40% for BCL6 (AUC, 0.702; sensitivity, 74.1%; specificity, 66.8%), 15% for p53 (AUC, 0.693; sensitivity, 64.8%; specificity, 71.5%), 35% at MUM1 (AUC, 0.726; sensitivity, 72.6%; specificity, 74.2%), 60% at c-Myc (AUC, 0.775; sensitivity, 79.6%; specificity, 75.3%), and 50% at Ki67 (AUC, 0.636; sensitivity, 64.9%; specificity, 67.3%). Immunohistochemical positivity for BCL2 was found in 66 (75.9%) patients, BCL6 in 69 (79.3%) patients, p53 in 38 (43.7%) patients, MUM1 in 50 (57.5%) patients, and c-Myc in 41 (47.1%) patients. EBV infection was detected by in situ hybridization analysis in 19 (21.8%) patients, while the Ki67 proliferative index was ≥50% in 56 (64.4%) patients (Table 3). There was a significant difference in recurrence rate according to p53 immunoreactivity (positive versus negative; *p* = 0.040), MUM1 (positive versus negative; *p* = 0.009), and c-Myc (positive versus negative; *p* = 0.027) (Table 3).

### 3.2. Follow-Up and Treatment Response

All patients completed the five cycles of HDMTX, and the mean follow-up duration was 60.2 months (range, 6–150 months). After induction therapy with HDMTX-based combination chemotherapy, 68 (78.2%) patients achieved CR and 18 (20.7%) patients achieved PR; the objective response rate was 98.9%, and only one patient had SD. Therefore, the 68 patients without remnant tumors underwent WBRT at a dose of 36 Gy, and the remaining 19 with remnant tumors underwent whole brain radiotherapy at a dose of 45 Gy. At 2 weeks after the WBRT, none of the remnant tumors were visible on MRI. An additional two cycles of cytarabine chemotherapy were administered to 19 patients who had remnant tumors after induction therapy with the HDMTX-based combination chemotherapy. There were no cases of PD during treatment.

### 3.3. PFS and OS

**Figure 1:**
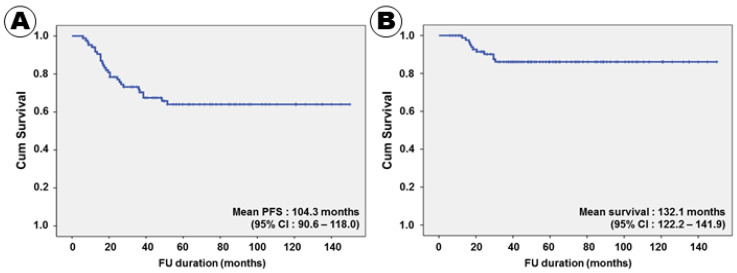
Kaplan–Meier survival curves for progression-free (**A**) and overall (**B**) survival.

Twenty-eight (32.2%) patients experienced recurrence during the follow-up period. The mean time to recurrence was 21.8 months, and the mean PFS was 104.3 months (95% confidence interval (CI), 90.6–118.0) (Figure 1A). One patient who did not respond to induction treatment with HDMTX experienced recurrence immediately after the end of the cytarabine chemotherapy despite the tumor having disappeared after WBRT; the time to recurrence was 6 months. In the univariate analysis, a normal serum LDH (*p* = 0.008), an ECOG performance score of 2–3 (*p* = 0.002), lower risk IELSG group (*p* < 0.001), no CSF seeding (*p* = 0.013), homogeneous enhancement on MRI (*p* = 0.034), gross total tumor resection (*p* = 0.004), negative immunoreactivity of BCL2 (*p* = 0.011), negative immunoreactivity of p53 (*p* = 0.014), negative immunoreactivity of MUM1 (*p* = 0.002), and negative immunoreactivity of c-Myc (*p* = 0.008) were associated with long PFS (Table 4).

Eleven (12.6%) patients succumbed to PCNSL; the mean OS was 132.1 months (95% CI, 122.2–141.9) (Figure 1B). The 3- and 5-year survival rates were 92.0% and 87.4%, respectively. All patients treated with the RTOG 9310 protocol survived for 12 months or more after receiving the PCNSL diagnosis. The shortest survival duration was 12.5 months. In the univariate analysis, a normal serum LDH level (*p* = 0.002), an ECOG performance score of 2–3 (*p* = 0.001), lower risk IELSG group (*p* = 0.001), unifocal tumor pattern (*p* = 0.017), no CSF seeding (*p* = 0.005), homogeneous enhancement on MRI (*p* = 0.004), gross total tumor resection (*p* = 0.003), negative immunoreactivity of BCL2 (*p* < 0.001), negative immunoreactivity of p53 (*p* = 0.003), negative immunoreactivity of MUM1 (*p* = 0.001), and negative immunoreactivity of c-Myc (*p* < 0.001) were associated with long PFS (Table 4).

### 3.4. Multivariate Analysis for Predicting Factors of PFS and OS

**Figure 2:**
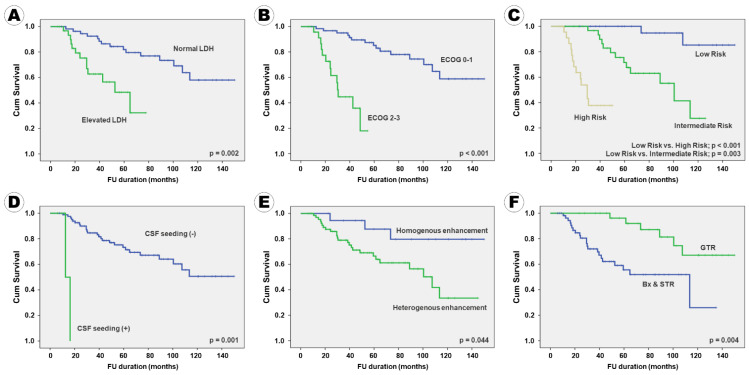

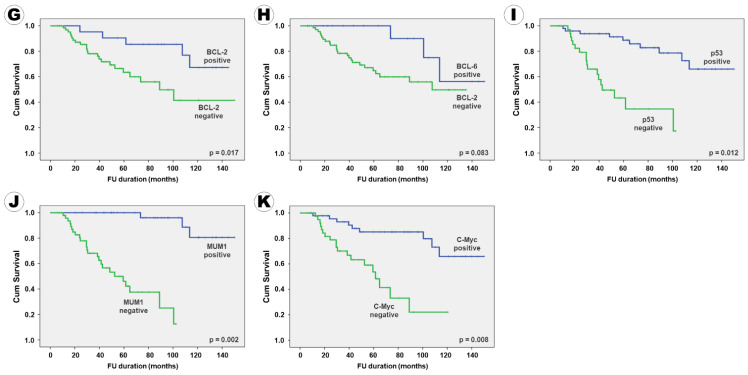
Kaplan–Meier survival curves for progression-free survival according to the clinical, radiological, and pathological factors: (**A**) serum LDH level, (**B**) ECOG performance score, (**C**) IELSG risk group, (**D**) CSF seeding, (**E**) enhancement pattern, (**F**) surgical extent, (**G**) BCL2 expression, (**H**) BCL6 expression, (**I**) p53 expression, (**J**) MUM1 expression, and (**K**) c-Myc expression.

In terms of PFS, 10 factors, namely serum LDH level; ECOG performance score; IELSG risk group; CSF seeding; enhancement pattern on MRI; surgical extent; immunoreactivity of BCL2, p53, MUM1, and c-Myc were positively associated with PFS in the univariate analysis. Six factors, including patient age, ocular involvement, tumor pattern, tumor size, BCL6 immunoreactivity, and Ki67 proliferative index, which tended to be associated with PFS, were included in the multivariate analysis. In conclusion, the following 11 factors were independently associated with PFS: serum LDH level; ECOG performance score; IELSG risk group; CSF seeding; enhancement pattern on MRI; extent of resection; BCL2 expression; BCL6 expression; p53 expression; MUM1 expression; c-Myc expression (Table 5). A Kaplan–Meier survival curve analysis for PFS, according to various factors, showed the same results (Figure 2).

**Figure 3:**
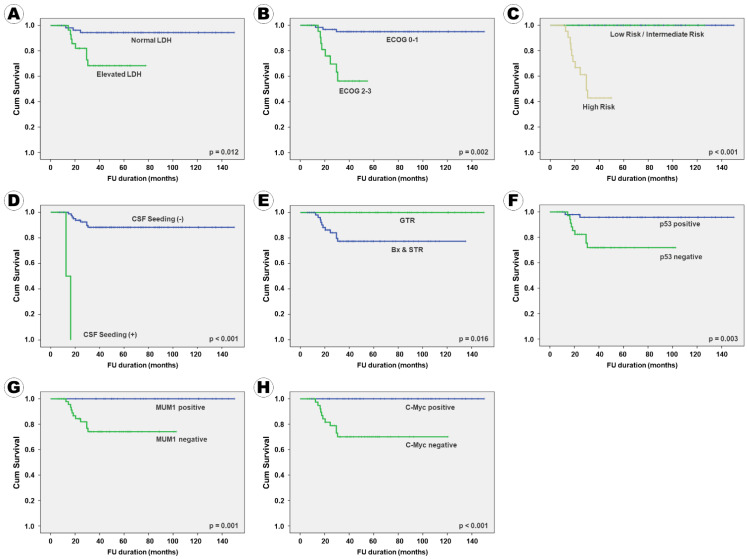
Kaplan–Meier survival curves of overall survival according to the clinical, radiological, and pathological factors: (**A**) serum LDH level, (**B**) ECOG performance score, (**C**) IELSG risk group, (**D**) CSF seeding, (**E**) surgical extent, (**F**) p53 expression, (**G**) MUM1 expression, and (**H**) c-Myc expression.

In terms of OS, 11 factors, namely serum LDH level; ECOG performance score; IELSG risk group; tumor pattern; CSF seeding; enhancement pattern on MRI; surgical extent; BCL2, p53, MUM1, and c-Myc immunoreactivity were positively associated with PFS in the univariate analysis. Three factors, including ocular involvement, deep brain involvement, and BCL6 immunoreactivity, which tended to be associated with PFS, were included in the multivariate analysis. Additionally, patient age and Ki67 proliferative index were included in the multivariate analysis because they are known to be associated with the clinical outcome of PCNSL patients, while evidence for Ki67 can be found in the literature. The following eight factors were independently associated with OS: serum LDH level, ECOG performance score, IELSG risk group, CSF seeding, resection extent, p53 expression, MUM1 expression, and c-Myc expression (Table 5). These eight factors were also independently associated with PFS. A Kaplan–Meier survival curve analysis for OS according to various factors showed the same results (Figure 3).

### 3.5. Adverse Effects of RTOG 9310 Protocol for PCNSL Patients

A total of 87 patients completed the five-cycle HDMTX-based combination chemotherapy; among them, 101 adverse effects occurred in 38 patients during the 435 treatment cycles (Supplementary Table S2). The most common side effects were hematologic disturbances such as thrombocytopenia and leukopenia. Among these 101 adverse effects, 100 (99.0%) were tolerable at grades 1 and 2. Only one patient had a life-threatening adverse effect (grade 4 acute renal failure). As renal function recovered after hemodialysis and medical treatment, the two remaining cycles were successfully completed. Fifteen grade 2 adverse effects occurred in eight patients, who continued treatment with a dose reduction of HDMTX.

## 4. Discussion

As the present study was retrospective and performed at a single institute, the value of these results cannot be considered highly significant. However, a relatively large number of patients were treated with a homogeneously consistent protocol, and excellent outcomes were reported compared with previous data from the original RTOG report. Although the clinical results were somewhat different, the treatment methods had many similarities.

In terms of common points between the two studies, the treatment protocol was based on HDMTX plus procarbazine and vincristine. The regimen dosages and number of cycles were identical. In addition, the objective response rate after induction chemotherapy using HDMTX-based combination chemotherapy was similar between the two reports (98.9% in our report and 94% in the original RTOG report) [12]. However, there were several important differences in the clinical outcomes between the two studies. First, the OS was much longer in our study (mean, 132.1 months; median not reached) than that of the original RTOG report (median, 36.9 months). Additionally, the 3- and 5-year survival rates were much higher in our study versus the RTOG study (92.0% vs 52% and 87.4% vs 32%, respectively) [12]. Second, PFS was also much longer in our study than the RTOG study (mean, 104.3 months; median not reached vs median, 24.0 months, respectively) in the original RTOG study. In addition, the 3- and 5-year PFS rates were significantly higher in our study versus the original RTOG study (70.4% vs 41% and 63.5% vs 25%, respectively) [12]. Third, the original RTOG study reported that 53% of patients experienced grade 3 or 4 toxicity during induction treatment using HDMTX-based combination chemotherapy [12] versus only one patient (1.1%) in our study.

Several reasons can be considered for the explanation of different outcomes between our report and the original RTOG report. First, our study had an extremely low rate of adverse effects compared with the original RTOG study (53% vs 1.1% cases of grade 3 or 4 toxicity, respectively). HDMTX is known to be highly toxic to the kidneys and nervous system [12,21]. To reduce the toxicity of HDMTX, the RTOG protocol recommends that methotrexate 2.5 g/m^2^ be diluted into 500 mL of D5W solution plus 25 mEq NaHCO_3_ and infused over 2 h, while leucovorin 20 mg be administered parenterally every 6 h for 12 doses starting 24 h after the systemic HDMTX infusion starts. Leucovorin rescue is important for reducing the adverse effects of HDMTX, and its dose titration should be determined according to the MTX serum level. However, it was practically impossible for our institute to receive a laboratory report of serum MTX levels within 48 h after blood sampling due to our laboratory system. As we could not deliver the optimal leucovorin rescue, the strict alkalinization of urine was targeted with an intravenous NaHCO_3_ infusion. After the occurrence of grade 4 toxicity due to inappropriate leucovorin rescue in the early period of PCNSL treatment at our institute using RTOG protocol 9310, we began to control the urine pH at over 7.0 using the dynamic dose of NaHCO_3_ infusion mixed in D5W solution. Thereafter, there were no cases of grade 3–4 toxicity during induction chemotherapy using HDMTX. Without serious treatment-induced toxicity, most patients could finish the five cycles of HDMTX-based combination chemotherapy.

Second, the modification radiotherapy doses and fractionations differed between the studies. The initial RTOG report planned WBRT for a total dose of 45 Gy in 1.8-Gy fractions. Approximately halfway through the original RTOG study, evidence was growing from the single-institution experience that long-term survivors of combined HDMTX-based combined chemotherapy and cranial irradiation were developing permanent severe neurotoxicity [13]. The study was then modified so that those patients who achieved a CR at the end of the five cycles of HDMTX-based combination chemotherapy would receive a hyperfractionated WBRT course for a total dose of 36 Gy given in 1.20-Gy fractions twice-daily for 15 days; the twice-daily RT doses were separated by a minimum of 6 h. However, all patients in our study underwent WBRT with a reduced dose of 36 Gy in 1.8-Gy fractions for 20 days when no remnant tumor was detected after induction chemotherapy with HDMTX-based combination chemotherapy. A relatively large number of patients (78.2%) underwent WBRT with a reduced dose of 36 Gy in 1.8-Gy fractions for 20 days compared with the original RTOG report. Although our study did not show any data on the decline of cognitive function after treatment due to its short follow-up duration, modification of the WBRT according to the presence of remnant tumor could reduce the adverse effects of therapy.

Third, consolidation chemotherapy using cytarabine arabinoside was not used to treat patients who had no remnant tumor after induction chemotherapy and WBRT. The original RTOG protocol recommends that all patients receive two courses of high-dose cytarabine at the completion of cranial irradiation. Each course includes two doses of cytarabine 3 g/m^2^/day infused over 3 h and administered 24 h apart. All patients in the original RTOG study were treated with high-dose cytarabine chemotherapy, while only 19 (21.8%) received high-dose cytarabine chemotherapy in our study because tumors remained after induction chemotherapy and WBRT. However, there was no difference in recurrence rate between patients who were and those who were not treated with high-dose cytarabine chemotherapy, which suggested that it is possible for patients without remnant tumor after induction chemotherapy to not be treated with consolidation chemotherapy with high-dose cytarabine.

The present study highlights the importance of a good patient condition during treatment as a major factor for predicting clinical outcomes. Our minimal modification of the RTOG protocol, such as maintaining a urine pH over 7.0, during HDMTX treatment, the application of WBRT dose and fraction according to remnant tumor status after induction chemotherapy, and the omission of consolidation chemotherapy with high-dose cytarabine, could reduce the adverse effects. As a result, patients successfully completed the full treatment cycles, which improved their clinical outcomes. In fact, our study showed that good patients’ performance status estimated by the ECOG performance score, which is commonly applied to other cancer patients, is a strong prognostic factor.

Although the original RTOG report did not show any pathological information associated with clinical outcomes, several studies have suggested that certain biomarkers are associated with prognosis. Among them, BCL2 and BCL6 have been studied worldwide for their prognostic role in PCNSL [17,22,23,24]. Although our study findings suggested that immunohistochemical positivity for BCL2 and BCL6 should be independently associated with a short PFS rather than OS in a multivariate analysis, controversy persists regarding the role of BCL2 and BCL6 protein expression on the prognosis of PCNSL patients. It is unclear whether the inconclusive findings across BCL2 studies were caused by different cutoffs used to define its positivity, which ranged from 50% to 70% [25,26,27,28]. However, Tapia et al. evaluated several cutoffs to define BCL2 positivity (30%, 50%, and 70%) and found no significant association between BCL2 expression and PCNSL patient prognosis [29]. Therefore, further comprehensive studies are essential to determine the prognostic role of BCL2 and BCL6 in PCNSL patients.

In addition, c-Myc is a widely studied biomarker in PCNSL with DLBCL cells. The expression of c-Myc proteins has been described in up to 70–90% of PCNSL DLBCL cases [30,31]. Although systemic DLBCL with high c-Myc expression has been associated with a worse prognosis, the findings were inconclusive for PCNSL with DLBCL cells [21]. Interestingly, c-Myc gene rearrangements occur at significantly lower frequencies (3–8%) [30,32], which suggests that increased c-Myc protein expression might be attributable to other genetic aberrations, such as *MYC* gene mutations or altered regulation of expression (e.g., through epigenetic modifications) independent of *MYC* gene rearrangements [21]. Although our study findings suggested that a high expression of c-Myc is strongly associated with PFS and OS with a cutoff value of 60%, there are no conclusive findings regarding the prognostic role of c-Myc in PCNSL patients. Several studies have shown that c-Myc protein overexpression (defined as immunohistochemical staining with at least 40% tumor cells) was associated with worse OS and PFS [26,27,33], but other studies found no significant difference in prognosis [25,30]. It is important to note that these studies used the same cutoff to define positive c-Myc protein expression. Our cutoff value of c-Myc expression in immunohistochemical staining was 60%; Tapia et al. used a cutoff value of 30%, which was relatively low compared with ours, to define c-Myc positivity and reported an association between c-Myc expression and poor outcome [29]. It remains unclear why these results have varied across studies, and it does not seem to be related to the c-Myc antibodies used.

With minimal modification of the original RTOG 9310 protocol for treating PCNSL, our study showed much improved clinical outcomes compared with the original RTOG report and suggested that several pathological biomarkers were associated with the prognosis of PCNSL patients treated with the RTOG 9310 protocol. However, several limitations must be noted. First, inherent bias was introduced by the retrospective nature of the study. We attempted to reduce this bias by collecting patient data from complete medical and radiological records and recruiting patients treated using the single and consistent RTOG 9310 protocol. Although multiple investigators without patient information independently reviewed the pathological slides and radiological images, we cannot clearly claim that no bias originated from this retrospective study. Despite these efforts, however, the conclusions drawn here require further validation through prospective and randomized clinical trials.

Second, although two different investigators examined and recorded the immunohistochemical staining data in the pathological report of the samples, it is uncertain whether the immunohistochemical results obtained in the pathological reports were correct because the assessment of immunohistochemical staining results is qualitative and often subjective. Reasonable run-to-run reproducibility with repeated reviews of pathological slides is essential for the proper implementation of these cutoff levels. In addition, threshold levels require adjustments according to the sensitivity of the method used. For this reason, we used specificity–sensitivity testing to determine the optimal cutoff level. However, additional studies are necessary to validate the reproducibility of our immunohistochemical staining method.

Third, our study used the traditional treatment protocol, which has not been updated for 13 years. New treatment strategies, such as combination therapy with rituximab and temozolomide, have recently evolved to achieve a median OS of 90 months [21]. Although several clinical trials are also investigating the role of induction rituximab in newly diagnosed PCNS DLBCL randomized to rituximab, MTX, teniposide, Bis-chlorethylnitrosourea (BCNU), prednisolone (R-MBVP), or MBVP alone [24], limited PCNSL patients are treated with new regimens rather than MTX in Korea because it lacks National Health Insurance approval. Therefore, combination treatment based on HDMTX is still generally considered the first-line treatment for PCNSL patients in Korea. However, with the accumulation of experience treating PCNSL patients with HDMTX, clinical outcomes have improved to as long as an OS of 131.1 months, while the adverse effects have also significantly reduced.

Finally, our study did not show the results of molecular genetic analyses, which has been updated and proven to have a prognostic role in PCNSL patients. For example, classic mutation profiles including MYD88 L265P and CD79B driving oncogenic toll-like receptor signaling, *BCL2* and *MYC* rearrangements in nodal/systemic activated B-cell–like lymphoma-type DLBCL, nuclear factor kappa-β signaling pathway dysregulation, and copy number variations have been shown to play an oncogenic function in PCNSL [34]. Comprehensive genetic and epigenetic research is required to prove the role of several molecular markers in predicting the prognosis of patients with PCNSL.

## 5. Conclusions

The present study investigated the PFS and OS of PCNSL patients who were treated with the RTOG 9310 protocol, which consists of HDMTX-based combination chemotherapy followed by WBRT. Despite the traditional protocol using HDMTX as the standard, the clinical outcomes were much better than those of other reports of recently updated treatment regimens. The minimal modification of the protocol through our accumulated experience of HDMTX-based combination chemotherapy reduced the serious adverse effects, which helped patients complete the full cycles of induction chemotherapy and consolidation radiotherapy. As a result, successful treatment achieved better outcomes in patients with PCNSL. However, further prospective and randomized clinical trials are necessary to validate our results.

## Figures and Tables

**Table 1:** Clinical features of 87 patients with primary central nervous system lymphoma.

Features	Total (*n* = 87)	Recurrence (+) (*n* = 28)	Recurrence (−) (*n* = 59)	*p* Value
Age				0.068
<60 years	49 (56.3%)	10 (20.4%)	39 (79.6%)	
≥60 years	38 (43.7%)	18 (47.7%)	20 (52.6%)	
Gender				0.923
Male	45 (51.7%)	14 (31.1%)	31 (68.9%)	
Female	42 (48.3%)	14 (33.3%)	28 (66.7%)	
Ocular involvement				0.917
Yes	6 (6.9%)	2 (33.3%)	4 (66.7%)	
No	81 (93.1%)	26 (32.1%)	55 (67.9%)	
Elevated serum LDH				0.132
Yes	32 (36.8%)	13 (40.6%)	19 (59.4%)	
Mo	55 (63.2%)	15 (27.3%)	40 (72.7%)	
Elevated protein in CSF				0.426
Yes	54 (62.1%)	19 (35.2%)	35 (64.8%)	
No	33 (37.9%)	9 (27.3%)	24 (72.7%)	
ECOG performance score				0.037
0–1	62 (71.3%)	15 (24.2%)	47 (75.8%)	
2–3	25 (28.7%)	13 (52.0%)	12 (48.0%)	
Risk of IELSG				0.048
Low (0–1)	28 (32.2%)	2 (7.1%)	26 (92.9%)	
Intermediate (2–3)	34 (39.1%)	13 (38.2%)	21 (61.8%)	
High (4–5)	25 (28.7%)	13 (52.0%)	12 (48.0%)	
Adjuvant cytarabine treatment				0.602
Yes	19 (21.8%)	7 (36.8%)	12 (63.2%)	
No	68 (78.2%)	21 (30.8%)	47 (69.2%)	

Abbreviations: CSF, cerebrospinal fluid; ECOG, Eastern Cooperative Oncology Group; IELSG, International Extranodal Lymphoma Study Group; LDH, lactate dehydrogenase.

**Table 2:** Radiologic features of 87 patients with primary central nervous system lymphoma.

Features	Total (*n* = 87)	Recurrence (+) (*n* = 28)	Recurrence (−) (*n* = 59)	*p* Value
Patterns				0.674
Unifocal	57 (65.5%)	17 (29.8%)	40 (70.2%)	
Multifocal	30 (34.5%)	11 (36.7%)	19 (63.3%)	
Deep brain involvement				0.392
Yes	38 (43.7%)	14 (36.8%)	24 (63.2%)	
No	49 (56.3%)	14 (28.6%)	35 (71.4%)	
CSF seeding				0.882
Yes	5 (5.7%)	2 (40.0%)	3 (60.0%)	
No	82 (94.3%)	26 (31.7%)	56 (68.3%)	
Maximal size of tumor				0.071
≥3 cm	40 (46.0%)	18 (45.0%)	22 (55.5%)	
<3 cm	47 (54.0%)	10 (21.3%)	37 (78.7%)	
Peritumoral edema				0.338
≥2 cm	33 (37.9%)	13 (39.4%)	20 (60.6%)	
<2 cm	54 (62.1%)	15 (27.8%)	39 (72.2%)	
Enhancement patterns				0.042
Homogeneous	19 (21.8%)	3 (15.8%)	16 (84.2%)	
Heterogeneous	68 (78.2%)	25 (36.8%)	43 (63.2%)	
Surgical extent				0.036
Gross total resection	31 (35.6%)	6 (19.4%)	25 (80.6%)	
Biopsy and partial resection	56 (64.4%)	22 (39.3%)	34 (60.7%)	

Abbreviations: CSF, cerebrospinal fluid.

**Table 3:** Histopathological features of 87 patients with primary central nervous system lymphoma.

Features	Total (*n* = 87)	Recurrence (+) (*n* = 28)	Recurrence (−) (*n* = 59)	*p* Value
Pathological classification				0.821
Diffuse large B-cell	74 (85.1%)	24 (32.4%)	50 (67.6%)	
Others *	13 (14.9%)	4 (30.8%)	9 (69.2%)	
Molecular subgroup				0.672
GCB	32 (36.8%)	12 (37.5%)	20 (62.5%)	
ABC	55 (63.2%)	16 (29.1%)	39 (70.9%)	
Bcl-2				0.188
Positive	66 (75.9%)	23 (34.8%)	43 (65.2%)	
Negative	21 (24.1%)	5 (23.8%)	16 (76.2%)	
Bcl-6				0.078
Positive	69 (79.3%)	25 (36.2%)	41 (59.4%)	
Negative	18 (20.7%)	3 (16.7%)	15 (83.3%)	
p53				0.040
Positive	38 (43.7%)	18 (47.4%)	20 (52.6%)	
Negative	49 (56.3%)	10 (20.4%)	39 (79.6%)	
MUM1				0.009
Positive	50 (57.5%)	25 (50.0%)	25 (50.0%)	
Negative	37 (42.5%)	3 (8.1%)	34 (91.9%)	
c-Myc				0.027
Positive	41 (47.1%)	19 (46.3%)	22 (53.7%)	
Negative	46 (52.9%)	9 (19.6%)	37 (80.4%)	
EBV in situ hybridization				0.804
Positive	19 (21.8%)	6 (31.6%)	13 (68.4%)	
Negative	68 (78.2%)	22 (32.4%)	46 (67.6%)	
Ki67				0.226
≥50%	56 (64.4%)	21 (37.5%)	35 (62.5%)	
<50%	31 (35.6%)	7 (22.6%)	24 (77.4%)	

Abbreviations: ABC, activated B-cell–like lymphoma; BCL-2, B-cell lymphoma-2; BCL-6, B-cell lymphoma-6; EBV, Epstein-Barr Virus; GCB, Germinal center B-cell-like lymphoma; MUM1, multiple myeloma-1. * Others mean 10 ALK-negative anaplastic large cell lymphomas, 2 peripheral T-cell lymphomas, and 1 marginal T-cell lymphoma.

**Table 4:** Univariate analysis for predicting factors of progression-free and overall survival.

Factors	Mean PFS (month)	HR (95% CI)	*p* Value	Mean OS (month)	HR (95% CI)	*p* Value
Age < 60 years	122.5 (±7.7)	2.33 (0.82–3.74)	0.109	127.9 (±7.1)	2.05 (0.78–3.32)	0.309
≥60 years	66.5 (±6.5)			104.9 (±4.5)		
Gender Male	106.1 (±9.5)	1.98 (0.28–3.68)	0.988	127.6 (±7.6)	1.97 (0.75–3.19)	0.390
Female	99.4 (±8.7)			132.2 (±6.1)		
Ocular involve (+)	105.3 (±6.8)	1.87 (0.71–3.03)	0.172	134.4 (±4.9)	1.55 (0.64–2.46)	0.146
involve (−)	42.0 (±8.4)			41.9 (±8.4)		
Serum LDH increase (−)	116.6 (±7.1)	6.18 (4.00–8.36)	0.008	142.6 (±4.1)	7.14 (4.62–9.66)	0.002
increase (+)	50.5 (±5.2)			60.3 (±5.1)		
CSF protein increase (−)	112.2 (±10.3)	1.39 (0.80–1.97)	0.387	137.7 (±6.7)	1.88 (0.58–3.18)	0.360
increase (+)	93.3 (±8.0)			119.9 (±6.5)		
ECOG score 0–1	118.7 (±6.7)	8.50 (5.61–11.39)	0.002	145.5 (±3.7)	9.04 (6.27–11.81)	0.001
score 2–3	34.4 (±3.4)			40.4 (±3.9)		
IELSG risk Low	141.9 (±5.3)	13.42 (8.45–18.39)	<0.001	145.1 (±7.7)	9.53 (6.15–12.91)	0.001
Intermediate	89.3 (±7.0)	4.33 (2.64–6.02)	0.011	136.5 (±7.4)	6.55 (4.09–9.01)	0.006
High	31.8 (±3.3)			14.4 (±1.9)		
Adjuvant ARA-C Yes	117.0 (±2.5)	1.73 (0.75–2.71)	0.362	126.7 (±7.3)	1.69 (0.61–2.77)	0.324
No	101.0 (±7.2)			133.1 (±6.4)		
Pattern Unifocal	113.2 (±7.1)	2.01 (1.01–3.01)	0.053	145.4 (±3.2)	4.11 (2.94–5.28)	0.017
Multifocal	68.3 (±8.3)			73.4 (±8.3)		
Deep location involve (−)	109.9 (±8.5)	1.52 (0.69–2.35)	0.264	138.7 (±5.4)	1.48 (0.51–2.45)	0.126
(+)	90.0 (±9.1)			111.2 (±8.0)		
CSF seeding (−)	106.0 (±6.7)	4.43 (1.58–7.28)	0.013	135.0 (±4.7)	5.74 (3.61–7.87)	0.005
(+)	14.4 (±1.9)			14.4 (±1.9)		
Tumor size < 3 cm	121.1 (±7.8)	1.92 (0.94–2.89)	0.089	132.2 (±6.7)	1.08 (0.22–1.94)	0.980
≥3 cm	82.2 (±9.7)			127.4 (±7.3)		
Peritumoral edema < 2 cm	101.6 (±6.9)	1.30 (0.62–1.97)	0.401	120.9 (±5.4)	1.52 (0.60–2.44)	0.594
≥2 cm	95.7 (±11.0)			129.4 (±8.5)		
Enhance Homogenous	130.3 (±10.3)	3.35 (1.27–5.44)	0.034	140.2 (±5.4)	6.92 (4.05–9.79)	0.004
Heterogeneous	91.0 (±7.6)			14.4 (±1.9)		
Extent of surgery GTR	128.6 (±8.2)	7.12 (4.96–9.28)	0.004	142.3 (±6.1)	7.13 (4.68–9.58)	0.003
Bx	80.5 (±7.4)			14.4 (±1.9)		
Pathology DLBCL	103.9 (±7.3)	1.10 (0.43–1.77)	0.919	132.8 (±5.3)	1.21 (0.39–2.03)	0.720
Others	92.6 (±11.4)			103.7 (±11.1)		
Cell type Non-GCL	109.9 (±8.2)	1.57 (0.61–2.53)	0.304	134.8 (±5.8)	1.48 (0.63–2.33)	0.407
GCL	84.8 (±8.7)			107.7 (±7.5)		
BCL-2 Negative	123.9 (±8.4)	5.65 (3.08–8.21)	0.011	146.4 (±4.8)	9.74 (6.54–12.94)	<0.001
Positive	92.9 (±8.8)			14.4 (±1.9)		
BCL-6 Negative	128.1 (±9.9)	1.83 (0.87–2.79)	0.102	135.0 (±4.7)	2.33 (0.97–3.69)	0.084
Positive	91.4 (±6.7)			40.4 (±5.6)		
p53 Negative	124.5 (±6.9)	5.23 (2.21–8.24)	0.014	135.0 (±4.7)	7.50 (3.99–11.01)	0.003
Positive	58.2 (±6.8)			14.4 (±1.9)		
MUM1 Negative	140.9 (±4.8)	8.21 (4.92–11.51)	0.002	139.2 (±5.1)	10.22 (7.45–12.99)	0.001
Positive	59.1 (±5.5)			20.8 (±5.9)		
c-Myc Negative	124.5 (±7.3)	6.09 (2.38–9.81)	0.008	145.0 (±4.7)	14.51 (8.77–20.25)	<0.001
Positive	64.8 (±7.6)			14.4 (±2.6)		
EBV Negative	103.7 (±7.6)	1.23 (0.29–2.17)	0.871	133.1 (±5.6)	1.36 (0.55–2.17)	0.728
Positive	100.1 (±12.9)			120.5 (±10.3)		
Ki67 < 50%	120.4 (±9.6)	1.95 (0.96–2.93)	0.091	145.5 (±4.4)	1.54 (0.86–2.23)	0.246
≥50%	81.6 (±6.3)			101.0 (±5.7)		

Abbreviations: ARA-C, cytarabine arabinoside; BCL-2, B-cell lymphoma-2; BCL-6, B-cell lymphoma-6; Bx, biopsy; CI, confidence interval; CSF, cerebrospinal fluid; ECOG, Eastern Cooperative Oncology Group; EBV, Epstein-Barr Virus; GCL, germinal cell B-cell-like lymphoma; GTR, gross total resection; HR, hazard ratio; IELSG, International Extranodal Lymphoma Study Group; MUM1, multiple myeloma-1; LDH, lactate dehydrogenase; OS, overall survival; PFS, progression-free survival.

**Table 5:** Multivariate analysis for predicting factors of progression-free and overall survival.

Factors	Progression-Free Survival		Overall Survival	
	HR (95% CI)	*p* Value	HR (95% CI)	*p* Value
Age (<60 yrs vs. ≥60 yrs)	2.23 (0.96–3.49)	0.098	1.89 (0.86–2.92)	0.209
Ocular involvement (No vs. Yes)	2.07 (0.92–3.21)	0.123	2.35 (0.95–3.75)	0.084
Elevation of LDH (No vs. Yes)	4.32 (1.78–6.86)	0.017	2.76 (1.27–4.25)	0.043
ECOG score (0–1 vs. 2–3)	5.18 (2.31–8.04)	0.008	4.26 (2.45–6.07)	0.011
IELSG risk (Low vs. High)	7.64 (4.26–11.02)	<0.001	8.42 (5.09–11.76)	<0.001
(Intermediate vs. High)	7.05 (4.19–9.91)	0.002	8.13 (4.28–11.98)	0.002
Patterns (Unifocal vs. Multifocal)	1.94 (0.76–3.12)	0.184	2.47 (0.99–3.94)	0.053
Deep structure involvement (No vs. Yes)	N.A.		1.92 (0.97–2.87)	0.126
CSF seeding	4.85 (2.54–7.16)	0.002	5.22 (3.14–7.29)	0.006
Tumor size (<3 cm vs. ≥3 cm)	2.44 (0.98–3.89)	0.056	N.A.	
Enhancement (Homogenous vs. Heterogeneous)	2.73 (1.12–3.87)	0.047	2.23 (0.88–3.48)	0.091
Extent of resection (GTR vs. Bx)	3.04 (1.28–4.79)	0.036	2.69 (1.14–4.24)	0.048
BCL-2 (Negative vs. Positive)	2.95 (1.33–4.57)	0.042	1.74 (0.81–2.65)	0.164
BCL-6 (Negative vs. Positive)	2.88 (1.29–4.47)	0.045	1.58 (0.84–2.31)	0.229
p53 (Negative vs. Positive)	3.21 (1.18–5.24)	0.031	3.37 (1.66–5.08)	0.036
MUM-1 (Negative vs. Positive)	3.94 (2.17–5.71)	0.028	4.62 (2.51–6.73)	0.017
c-Myc (Negative vs. Positive)	3.51 (2.33–4.69)	0.035	4.08 (2.16–6.01)	0.024
Ki67 (<50% vs. ≥50%)	2.11 (0.88–3.32)	0.122	2.31 (0.85–3.77)	0.086

Abbreviations: BCL-2, B-cell lymphoma-2; BCL-6, B-cell lymphoma-6; Bx, biopsy; CI, confidence interval; CSF, cerebrospinal fluid; ECOG, Eastern Cooperative Oncology Group; GTR, gross total resection; HR, hazard ratio; IELSG, International Extranodal Lymphoma Study Group; MUM1, multiple myeloma-1; LDH, lactate dehydrogenase; N.A., not assessed.

## Data Availability

The data presented in this study are available on request from the corresponding author. The data are not publicly available due to sensitive clinical data involved.

## Supplementary Materials

The following are available online at https://www.mdpi.com/article/10.3390/curroncol28060393/s1, Table S1: Treatment flow chart of RTOG 9310 protocol for primary central nervous system lymphoma, Table S2: Common Terminology Criteria for Adverse Events in RTOG9310 protocol (total 435 cycles).
