# Supplementary material for: Clinical Outcomes of RTOG 9310 Protocol for Primary Central Nervous System Lymphoma: Single-Center Experience with 87 Patients
Source: Curr Oncol. 2021 Nov 12;28(6):4655–72. doi: 10.3390/curroncol28060393 (PMC8628744; doi:10.3390/curroncol28060393)
Supplement: Supplementary file 1 [file curroncol-28-00393-s001.zip › curroncol-1467234-supplementary.pdf]

Table S1. Treatment flow chart of RTOG 9310 protocol for primary central nervous system lymphoma.

	Weeks											
	1	2	3	4	5	6	7	8	9	10	11-15	16-19
Methotrexate IV 2.5g/m^2 once	√		√		√		√		√			
Vincristine IV 1.4mg/m^2 once	√		√		√		√		√			
Procarbazine PO 100mg/m^2/day for 7 days	√				√				√			
Methotrexate Intra-Ommaya 12mg		√		√		√		√		√		
Leucovorin 20mg QID for 12 doses	√		√		√		√		√			
Leucovorin 10mg BID for 12 doses		√		√		√		√		√		
Dexamethasone mg/day for 7 days	16	12	8	6	4	2						
Whole-brain radiotherapy											√	
Cytarabine 3g/m^2/day												√

***Abbreviations.** BID, every 12 hours; IV, intravenous injection; PO, parenteral administration; QID, every 6 hours.

Table S2. Common Terminology Criteria for Adverse Events in RTOG9310 protocol (total 435 cycles)

	Total	Grade 1 (mild)	Grade 2 (moderate)	Grade 3 (severe)	Grade 4-5 (life-threating)
Hematologic disorder[1)]	30	25	5	0	0
GI disorder[2)]	13	11	2	0	0
Nervous disorder[3)]	12	9	3	0	0
Vascular disorder[4)]	10	8	2	0	0
Hepatobiliary disorder[5)]	8	7	1	0	0
Psychiatric disorder[6)]	7	7	0	0	0
Ophthalmologic disorder[7)]	6	5	1	0	0
Musculoskeletal disorder[8)]	4	4	0	0	0
Skin problem[9)]	4	4	0	0	0
Immune disorder[10)]	3	3	0	0	0
Renal disorder[11)]	3	1	1	0	1
Endocrine disorder[12)]	1	1	0	0	0
Cardiac disorder	0	0	0	0	0
Ear and labyrinth disorder	0	0	0	0	0
Infections	0	0	0	0	0
Metabolic disorder	0	0	0	0	0
Respiratory disorder	0	0	0	0	0
Total	101	85	15	0	1

1) leukopenia and thrombocytopenia, 2) nausea and diarrhea, 3) dysesthesia and paresthesia, 4) pitting edema, 5) increased hepatic enzyme and serum bilirubin level, 6) confusion and delirium, 7) xerophthalmia, 8) fatigue and myalgia, 9) urticaria, 10) allergic reaction, 11) acute kidney injury and hematuria, 12) glucose intolerance.
